# Effects of doxofylline as an adjuvant on severe exacerbation and long‐term prognosis for COPD with different clinical subtypes

**DOI:** 10.1111/crj.13670

**Published:** 2023-08-10

**Authors:** Mei‐Feng Chen, Wei He, De‐Sheng Huang, Hui Jia, Zhao‐Shuang Zhong, Nan Li, Shan‐Shan Li, Shu‐Yue Xia

**Affiliations:** ^1^ Department of Respiratory and Critical Care Medicine Clinical Medical College and The First Affiliated Hospital of Chengdu Medical College Chengdu Sichuan China; ^2^ Department of Respiratory and Critical Care Medicine Central Hospital Affiliated To Shenyang Medical College Shenyang China; ^3^ Department of Public Health China Medical University Shenyang China

**Keywords:** COPD, different clinical subtypes, dose adjustment rate of glucocorticoid, doxofylline, severe acute exacerbations

## Abstract

**Objective:**

This study aimed to investigate the effectiveness of doxofylline as an adjuvant in reducing severe exacerbation for different clinical subtypes of chronic obstructive pulmonary disease (COPD).

**Methods:**

The clinical trial was an open‐label non‐randomized clinical trial that enrolled patients with COPD. The patients were divided into two groups (doxofylline group[DG] and non‐doxofylline group[NDG]) according to whether the adjuvant was used. Based on the proportion of inflammatory cells present, the patients were divided into neutrophilic, eosinophilic, and mixed granulocytic subtypes. The rates of severe acute exacerbation, use of glucocorticoids, and clinical symptoms were followed up in the first month, the third month, and the sixth month after discharge.

**Results:**

A total of 155 participants were included in the study. The average age of the participants was 71.2  ±  10.1 years, 52.3% of the patients were male, and 29.7% of the participants had extremely severe cases of COPD. In the third month after discharge the numbers of patients exhibiting severe exacerbation among the neutrophilic subtype were 5 (6.6%) in the DG versus 17 (22.4%) in the NDG (incidence rate ratio[IRR] = 0.4 [95% CI: 0.2–0.9] *P* = 0.024). In the sixth month after discharge, the numbers were 3 (3.9%) versus 13 (17.1%; IRR = 0.3 [95%; CI: 0.1–0.9], *P* = 0.045), and those for the eosinophilic subtype were 0 (0.0%) versus 4 (14.8%), *P* = 0.02. In the eosinophilic subtype, the results for forced expiratory volume in the first second and maximal mid‐expiratory flow were significantly higher in the DG. The mean neutrophil and eosinophil levels were significantly lower than in the NDG among the neutrophilic subtype, and the neutrophil percentage was lower than in the NDG among the eosinophilic subtype. At the six‐month follow‐up, the dose adjustment rates of the neutrophilic and eosinophilic subtypes showed a significant difference (*P*< 0.05).

**Conclusions:**

As an adjuvant drug, doxofylline has a good therapeutic effect on patients with the neutrophilic and eosinophilic clinical subtypes of COPD. It can reduce the incidence of severe exacerbation, the use of glucocorticoids, and inflammatory reactions in the long term (when used for a minimum of 3 months).

## Graphical abstract

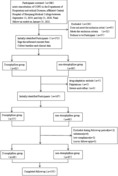

## 1 INTRODUCTION

Chronic obstructive pulmonary disease (COPD) is a common and high‐incidence disease affecting the respiratory system. At present, the prevalence rate of COPD among people over 40 years old in China is 13.7%,^1^ equating to about 100 million patients. The overall disease burden of COPD is ranked third among acute and chronic diseases, meaning that it represents a heavy burden to the social economy and public health globally.^2^ From continuous in‐depth research and developments in precision medicines, it has been realized that there are individual differences among patients with COPD in terms of, for example, susceptibility levels, exacerbation numbers, and lung function decline rates. In the past, pulmonary function was the core concern in the diagnosis and treatment of COPD; however, recent studies have found that forced expiratory volume in the first second (FEV_1_) alone cannot objectively reflect the complexity and heterogeneity of COPD.^3^, ^4^ The subtypes of COPD describe the disease attributes (single or multiple) among individual differences in patients, which are closely related to clinical prognosis (symptoms, acute exacerbation, response to treatment, disease progression rate, or time until death).^5^, ^6^ It is therefore suggested that subtypes should be taken into account to maximize the risk/benefit ratio of COPD treatment.

Bronchodilators and corticosteroids are the primary drugs used for the treatment of COPD at the acute exacerbation and stable stages, and they can be used independently or in combination. However, some patients have low sensitivity to corticosteroid therapy, meaning that using high doses can lead to decreased sensitivity and adverse reactions such as pneumonia and osteoporosis.^7^

Theophylline has been used in the treatment of COPD and asthma since 1937; however, because of the narrow safety treatment window, the GOLD Management Strategy guidelines recommended that it should only be used in patients who do not benefit from other bronchodilators and cannot afford treatment.^8^ Doxofylline is a new derivative of methylxanthine: Its pharmacological effect is so different from theophylline that it cannot simply be regarded as modified theophylline. It has no significant effect on any known phosphodiesterase isotype, no significant antagonistic effect on the adenosine receptors, and no direct effect on histone deacetylase, and it interacts with the β_2_‐adrenoceptor.^9^, ^10^, ^11^ At the same time, combining it with corticosteroids can increase sensitivity and reverse the corticosteroids' drug resistance.^12^ In the current context of the disease burden of COPD in China, doxofylline treatments are still widely used. However, at present, there is insufficient evidence regarding the effectiveness of doxofylline as an adjuvant on the deterioration, hospitalization rate, symptom improvement, and prognosis of different clinical subtypes of COPD. There are some studies in China, but the results are different from those in other countries, and the results are controversial. It is not yet clear whether doxofylline can be used as an adjuvant to the standard treatment of COPD, and there is a lack of domestic data on the clinical and economic benefits for patients who are unable to obtain adequate control from other pharmacological categories and have difficulty using medicine that must be inhaled.

The objective of this study was to compare the effectiveness of doxofylline in reducing severe acute exacerbation and improving long‐term prognosis in different clinical subtypes of COPD and to explore whether it can be used as an adjuvant for standard COPD treatments. Positive findings would indicate clinical and economic benefits for patients who cannot get enough control from other treatment options and find it difficult to use inhaled medicine and would provide a reference basis for the control of treatment drugs in the stable phase of COPD.

## 2 METHODS

### 2.1 Study design and oversight

G * Power was used to estimate the sample size, effect size *f* = 1, *α* = 0.05, 1 − *β* = 0.8. The estimated required sample size is 73, the lost rate was 5%, and the final sample size is 76. A total of 155 samples met the inclusion and exclusion criteria, and the sample size will continue to increase in the follow‐up study.

This clinical trial was an open‐label non‐randomized clinical trial that enrolled patients with acute exacerbation of COPD in the Department of Respiratory and Critical Diseases at the Central Hospital Affiliated to Shenyang Medical College between September 13, 2019, and July 31, 2020. The final follow‐up ended on January 31, 2021. The study comprised a 1‐week drug adaptation period followed by a treatment phase. The patients were divided into two groups (doxofylline group [DG]: *n* = 68; non‐doxofylline group [NDG]: *n* = 87) according to whether doxofylline was used in the treatment plan. After discharge, the DG continued to take doxofylline sustained‐release tablets (0.2 g bid, oral for 6 months) alongside inhaled drugs, whereas the NDG only used inhaled drugs. Adherence was assessed by counting the remaining pills at drug returns at the first‐, third‐, and sixth‐month follow‐ups. Baseline data were collected by face‐to‐face assessments conducted within 72 h of admission. The members of the research group, who each received the same rigorous training, were responsible for the follow‐ups with chronic disease management. The follow‐ups, including medication return and dispensing of new medication, were carried out at the dates of the first, third, and sixth months after discharge.

This study was conducted in line with the Helsinki Statement and approved by the Ethics Committee of Shenyang Medical College Hospital. All patients provided written informed consent before undertaking the study.

### 2.2 Participants

The inclusion criteria for participants were as follows: (1) aged 40–85 with a predominant respiratory diagnosis of COPD (FEV_1_/forced vital capacity [FVC] ratio of <0.7); (2) able to cooperate to complete the post‐bronchodilator spirometry; (3) able to understand and independently complete the COPD Assessment Test (CAT), the modified Medical Research Council (mMRC) Questionnaire, and related questionnaires after explanation by the investigators; and (4) willing to voluntarily participate in the study and sign the informed consent form. The exclusion criteria were as follows: (1) participating in other clinical studies; (2) chronic or acute respiratory diseases other than COPD, such as active pulmonary tuberculosis, lung tumor, interstitial pneumonia, and pleural effusion; (3) suffering from primary cardiovascular disease or severe liver, kidney, cerebrovascular, or hematological diseases; (4) suffering from a malignant tumor; (5) unclear consciousness, mental disorder, or neurological history and physical activity disorder; (6) allergic to doxofylline or xanthine derivatives; and (7) unwilling to cooperate, found it difficult to fill out the questionnaire, or unable to communicate at all. The withdrawal criteria were as follows: (1) failed to take medicine regularly as required; (2) serious adverse events occurred and patient should not continue to undergo the trial; and (3) subject asked to withdraw.

### 2.3 Clinical subtypes

The DG and the NDG were each divided into three clinical subtypes according to the type of internal inflammation: neutrophilic (neutrophil ≥ 61%, eosinophil < 2%), eosinophilic (eosinophil ≥ 2%, neutrophil < 61%), and mixed granulocytic (eosinophils > 2% + neutrophils > 61%/eosinophils < 2% + neutrophils < 61%).

### 2.4 Outcomes

The primary outcome was the number of participant‐reported severe exacerbations requiring hospital admission during the 6‐month treatment period. In addition to exacerbation data, the following secondary outcomes were collected: dose adjustment rate of inhaled drugs containing glucocorticoid; adverse events; clinical symptoms; COPD‐related health status (CAT scale, ≤5 being the norm for healthy nonsmokers and >30 indicating a very high effect of COPD on quality of life)^13^; mMRC dyspnea score (range: 0 [not troubled by breathlessness except on strenuous exercise] to 4 [too breathless to leave the house or breathless when dressing or undressing])^14^; inflammatory cells in serum (leukocyte, neutrophil percentage, lymphocyte percentage, eosinophil percentage); and changes in post‐bronchodilator spirometry. For all of these outcomes, the stable stage was compared with the sixth month after discharge. The classification for COPD severity was based on GOLD criteria.

### 2.5 Statistical methods

The analysis was carried out according to the intention‐to‐treat principle. A per‐protocol analysis, excluding participants classed as non‐adherent (<80% of doses taken), was performed to measure sensitivity. The primary clinical outcomes of the number of COPD exacerbations for each subgroup were compared using a negative binomial model with an appropriate dispersion parameter to adjust for inter‐participant variability. Estimates were adjusted for baseline covariates known to be related to outcome: age, smoking index, GOLD stage, number of exacerbations in the previous 1 year, body mass index, and baseline treatment for COPD. The subgroup analyses were undertaken by adding a treatment × variable interaction term to the model using the primary outcome. Analysis was performed using SPSS version 26.0. The clinical subtypes data of the DG and NDG were analyzed with mean ± SD (normal distribution) or median/interquartile (*n*, %), tested with the chi‐square test. The data with irregular variance and non‐normally distributed detailed range (non‐normal distribution) were tested by Mann–Whitney *U* and Kruskal–Wallis. Counting data were described by ratio or composition ratio, tested with the Mann–Whitney *U* or the Kruskal–Wallis test. A 5% two‐sided significance level was used throughout.

## 3 RESULTS

### 3.1 Participant characteristics

**FIGURE 1:**
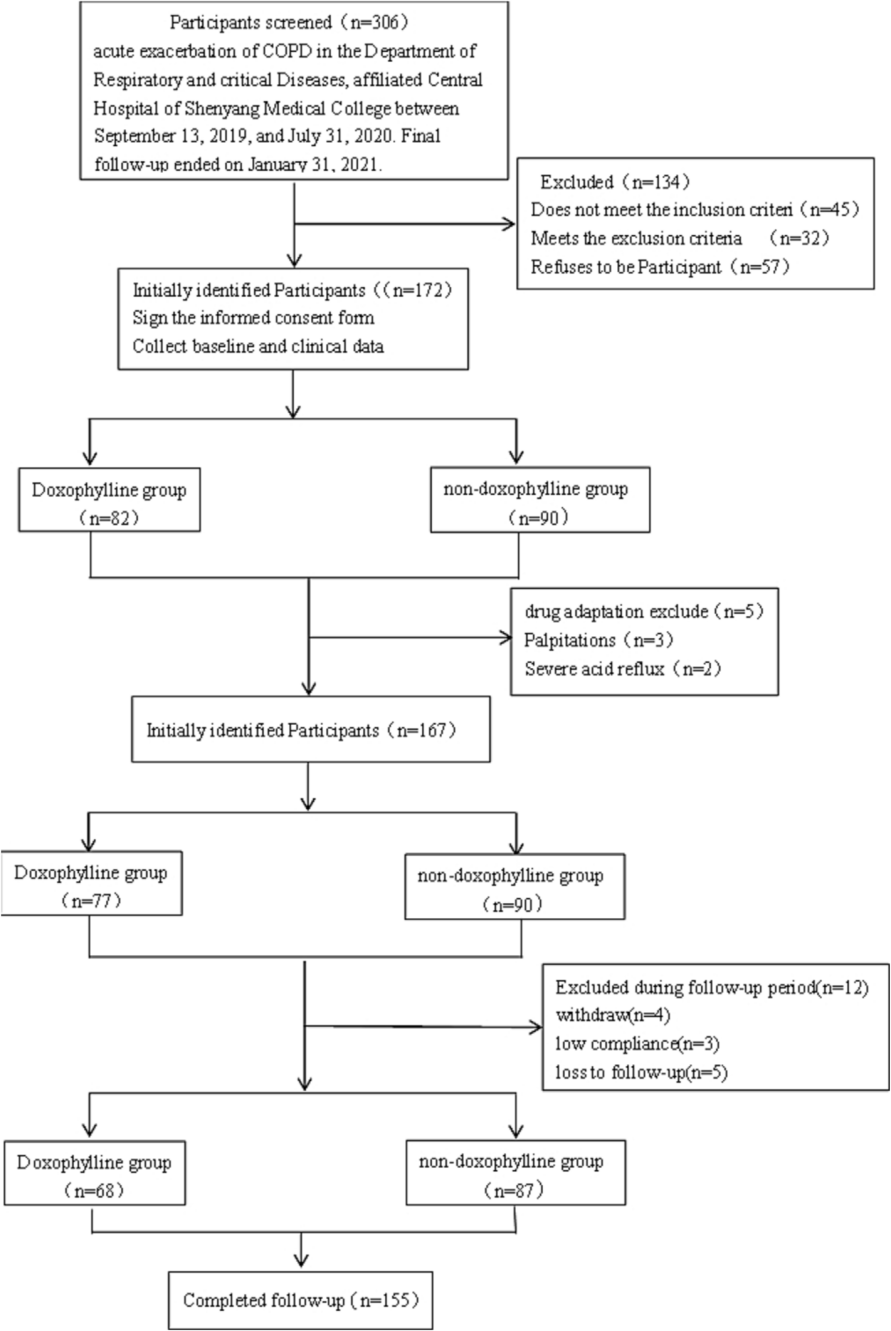
Participant participation flow chart.

A total of 172 participants were included: 82 in the DG and 90 in the NDG. During the 1‐week drug adaptation and follow‐up period, there were 17 further exclusions because of adverse reactions (*n* = 5), withdrawal from the study (*n* = 4), low compliance (*n* = 3), or loss to follow‐up (*n* = 5). A final total of 155 patients completed the study: 68 to the DG and 87 to the NDG. Participant involvement in the trial is outlined in Figure 1.

There were no clinically significant differences in baseline data characteristics between the DG and the NDG (Table 1). The mean age of the participants was 70.8 ± 10.8 years, 52.3% were male, the mean BMI was 22.6 kg/m^2^, and 56.1% were smokers. The mean age of smoking was 27.0 years, and the smoking index was 600. The mean length of diagnosis was 8 years. The most common complications were hypertension/coronary heart disease (69.7%), diabetes (16.8%), digestive system diseases (11.6%), cerebrovascular diseases (9.7%), and cor pulmonale (7.1%). According to FEV_1_ testing, the highest proportion of participants (38.0%) had severe COPD: In total, 29.7% had very severe COPD, 67.8% moderate to severe, and 2.6% mild. The CAT scores indicated that COPD was severely affecting participants' lives (mean [SD] = 22.6 ± 2.7). In terms of treatments, 81.6% of participants were using combination therapies of long‐acting muscarinic antagonists + ICS, long‐acting β2‐agonists + ICS, or long‐acting muscarinic antagonists + long‐acting β2‐agonists + ICS. The mean leukocyte level of the participants was 8.5 ± 3.5 × 10^9/L, and the mean percentages of neutrophil, lymphocytes, and eosinophil were 68.5%, 20.2%, and 1.5%, respectively. The participants were divided into three clinical subtypes according to the proportion of inflammatory cells present: neutrophilic (49.0%), eosinophilic (17.4%), and mixed granulocytic (33.5%). The participants' baseline information is presented in Table 1.

**TABLE 1:** Baseline characteristics of participants to doxophylline group and non‐doxophylline group.

Parameters	Non‐doxophylline group (*n* = 87)	Doxophylline group (*n* = 68)	Total	*P* value
Gender (*n*, %)				0.881
Male	45 (51.7%)	36 (52.9%)	81 (52.3%)	
Female	42 (48.3%)	32 (47.1%)	74 (47.7%)	
Age (year)^b^	72.5 ± 9.3	69.5 ± 10.3	71.2 ± 10.1	0.073
BMI (kg/m^2^)^b^	22.5	22.8	22.6	0.215
Smoking (*n*, %)	45 (51.7%)	42 (61.8%)	87 (56.1%)	0.211
Age of smoking	28.0	26.5	27.0	0.895
Smoking index^b^	700.0	600.0	600.0	0.149
Length of diagnosis^b^	8.0	9.0	8.0	0.899
Exacerbations in last 1 year	0.8 ± 0.7	0.8 ± 1.7	0.8 ± 1.2	0.887
Comorbidities (*n*, %)				
Hypertension/coronary heart disease	62 (71.3%)	46 (67.6%)	108 (69.7%)	0.627
Cor pulmonale	6 (6.9%)	5 (7.4%)	11 (7.1%)	0.913
Digestive system diseases	10 (11.5%)	8 (11.8%)	18 (11.6%)	0.958
Diabetes	15 (17.2%)	11 (16.2%)	26 (16.8%)	0.860
Cerebrovascular diseases	9 (10.3%)	6 (8.8%)	15 (9.7%)	0.751
GOLD stage (*n*, %)				
Level 1	2 (2.3%)	2 (2.9%)	4 (2.6%)	
Level 2	23 (26.4%)	14 (20.6%)	37 (23.9%)	0.371
Level 3	41 (47.1%)	27 (39.1%)	68 (43.9%)	
Level 4	21 (24.1%)	25 (36.8%)	46 (29.7%)	
FEV_1_% predicted^b^	41.0	36.0	38.0	0.091
MEF%^b^	29.0	27.0	27.0	0.075
mMRC dyspnea score (*n*, %)				
1: Breathless hurrying	2 (2.3%)	2 (2.9%)	4 (2.6%)	0.627
2: Slower than contemporaries	19 (21.8%)	15 (22.1%)	34 (21.9%)	
3: Stop after 100 m	44 (50.6%)	28 (41.2%)	72 (46.5%)	
4: Breathless leaving house	22 (25.3%)	23 (33.8%)	45 (29.0%)	
COPD assessment test scole (CAT)	22.5 ± 2.8	22.7 ± 2.6	22.6 ± 2.7	0.615
GOLD treatment (*n*, %)				
LAMA only	16 (18.4%)	14 (20.6%)	30 (19.4%)	0.114
LAMA + ICS	38 (43.7%)	32 (47.1%)	70 (45.2%)	0.089
LABA + ICS	13 (14.9%)	8 (11.8%)	21 (13.5%)	0.916
LAMA + LABA + ICS	20 (23.0%)	14 (20.6%)	34 (21.9%)	0.181
Leukocyte^a^ (10^9/L)	8.6 ± 3.8	8.4 ± 3.2	8.5 ± 3.5	0.984
Neutrophil percentage^b^ (%)	69.7	67.7	68.5	0.580
Lymphocyte percentage^b^ (%)	20.2	20.5	20.2	0.892
Eosinophil percentage^b^ (%)	2.2	1.5	1.5	0.445
Clinical phenotypes (*n*, %)				0.346
Neutrophilic	43 (49.4%)	33 (48.5%)	76 (49.0%)	
Eosinophilic	12 (13.8%)	15 (22.1%)	27 (17.4%)	
Mixed granulocytic	32 (36.8%)	20 (29.4%)	52 (33.5%)	
Combined with rheumatological conditions (*n*, %)	0 (0.0%)	0 (0.0%)	0 (0.0%)	>0.999
Mandated use of corticosteroids or recently corticosteroids used	0 (0.0%)	0 (0.0%)	0 (0.0%)	>0.999

*Note*: Counting data are expressed in frequency and percentage (*n*, %).

^a^ Mean ± SD.

^b^ Median (interquartile range).

### 3.2 Outcomes for different clinical subtypes in doxofylline group and non‐doxofylline group, per‐protocol population

**FIGURE 2:**
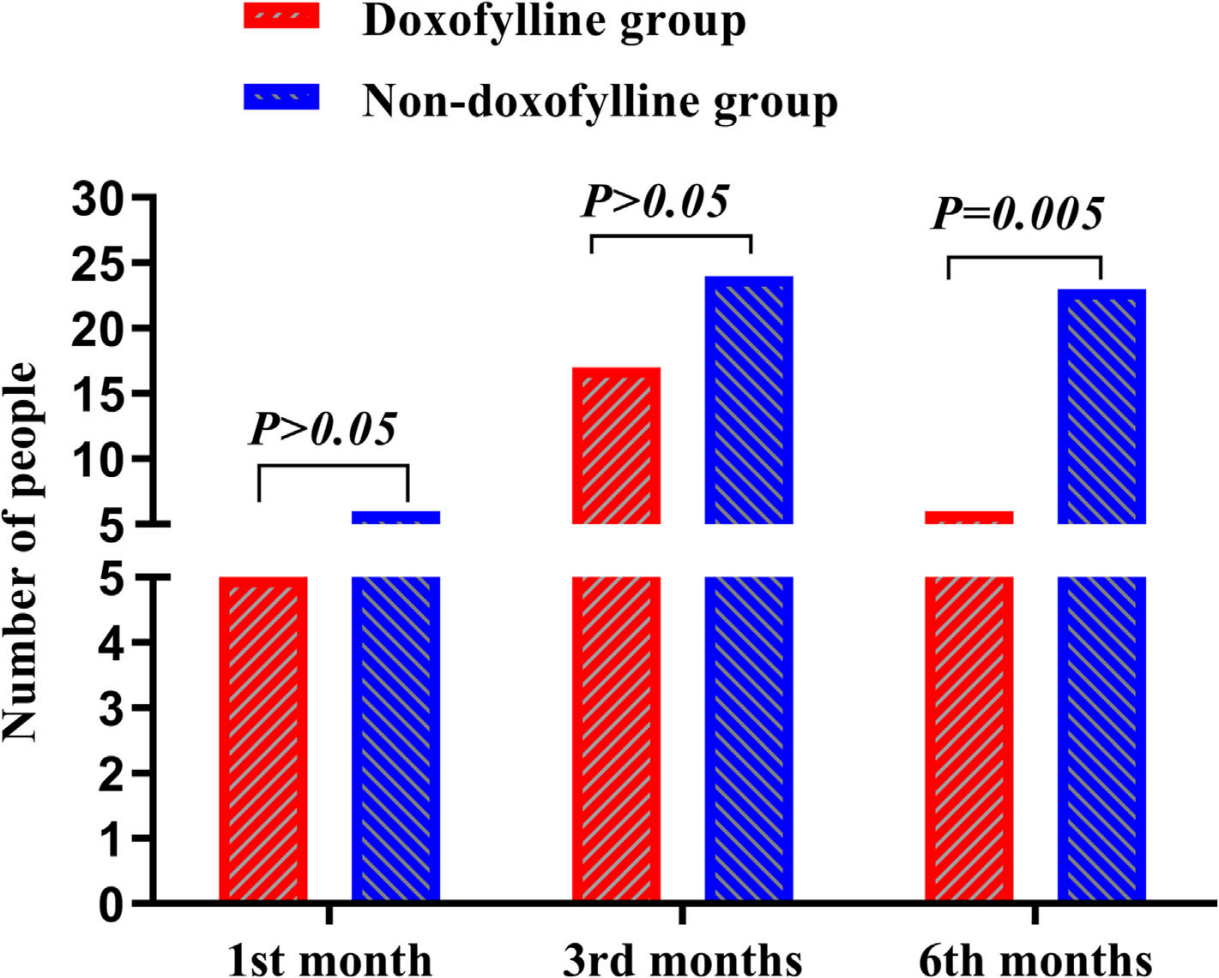
The outcomes for the participants in the doxofylline group (DG) and the non‐doxofylline group (NDG).

Primary outcome (severe exacerbation) data for all 6 months of follow‐up were available for all 155 participants (68 in the DG; 87 in the NDG) (Table 2). In total, there were 81 exacerbations: 28 in the DG and 53 in the NDG. In the DG, 11 of the exacerbations were in the neutrophilic subtype, five in the eosinophilic, and 12 in the mixed granulocytic. In the NDG, 33 were neutrophilic, five were eosinophilic, and 15 were mixed granulocytic. There was no significant difference between the numbers of acute exacerbations for the neutrophilic, eosinophilic, or mixed granulocytic subtypes in the first month after discharge. In the third month after discharge, there was a clinically significant difference (*P* = 0.024) in the neutrophilic subtype, with five exacerbations in the DG (6.6%) compared with 17 (22.4%) in the NDG (incidence rate ratio [IRR] = 0.4; 95% CI: 0.2–0.9), but there were no significant differences in the eosinophilic and mixed granulocytic subtypes. In the sixth month after discharge, there were clinically significant differences in the neutrophilic subtype (*P* = 0.045) (three exacerbations in the DG [3.9%] compared with 13 [17.1%] in the NDG; IRR = 0.3 [95% CI: 0.1–0.9]) and the eosinophilic subtype (*P* = 0.028) (0 exacerbations in the DG [0.0%] compared with four [14.8%] in the NDG), and there was no significant difference in the mixed granulocytic subtype. In addition, there was no statistical difference in the incidence of adverse events. The outcomes for the participants in the DG and the NDG are presented in Table 2 and Figure 2.

**TABLE 2:** Outcomes for participants to doxophylline group and non‐doxophylline group, per‐protocol population.

Parameters	Doxophylline group (*n* = 68)	Non‐doxophylline group (*n* = 87)	Rate ratio (95% CI)	*P* value
Number of moderate or severe exacerbations (*n*, %)				
1 month	5 (3.2%)	6 (3.9%)	1.0 (0.3–3.4)	0.913
3 months	17 (11.0%)	24 (15.5%)	0.9 (0.5–1.5)	0.717
6 months	6 (3.9%)	23 (14.8%)	0.3 (0.1–0.7)	0.005
Time of moderate or severe exacerbations (*n*, %)				
1 month	0.1 ± 0.3	0.1 ± 0.3	0.1 ± 0.3	0.913
3 months	0.3 ± 0.4	0.3 ± 0.5	0.3 ± 0.4	0.718
6 months	0.1 ± 0.4	0.3 ± 0.6^a^	0.2 ± 0.5	0.006
Total time	0.4 ± 0.5	0.7 ± 0.6	0.6 ± 0.6	0.215
FEV_1_% predicted	44.5	46.0	46.0	0.508
MEF%	33.0	34.0	34.0	0.634
Leukocyte (10^9/L)‐6 months	7.0 ± 2.6	7.5 ± 2.4	7.2 ± 2.5	0.143
Neutrophil percentage (%)‐6 months	62.2	65.8^a^	63.7	0.002
Lymphocyte percentage (%)‐6 months	21.1	23.4	21.9	0.375
Eosinophil percentage (%)‐6 months	1.5	2.3	1.8	0.063
Adverse events rate (*n*, %)				0.628
Acid regurgitation	2 (2.9%)	0 (0.0%)		
Palpitation	3 (5.9%)	0 (0.0%)		

^a^ There was a clinically significant difference between DG and NDG.

**FIGURE 3:**
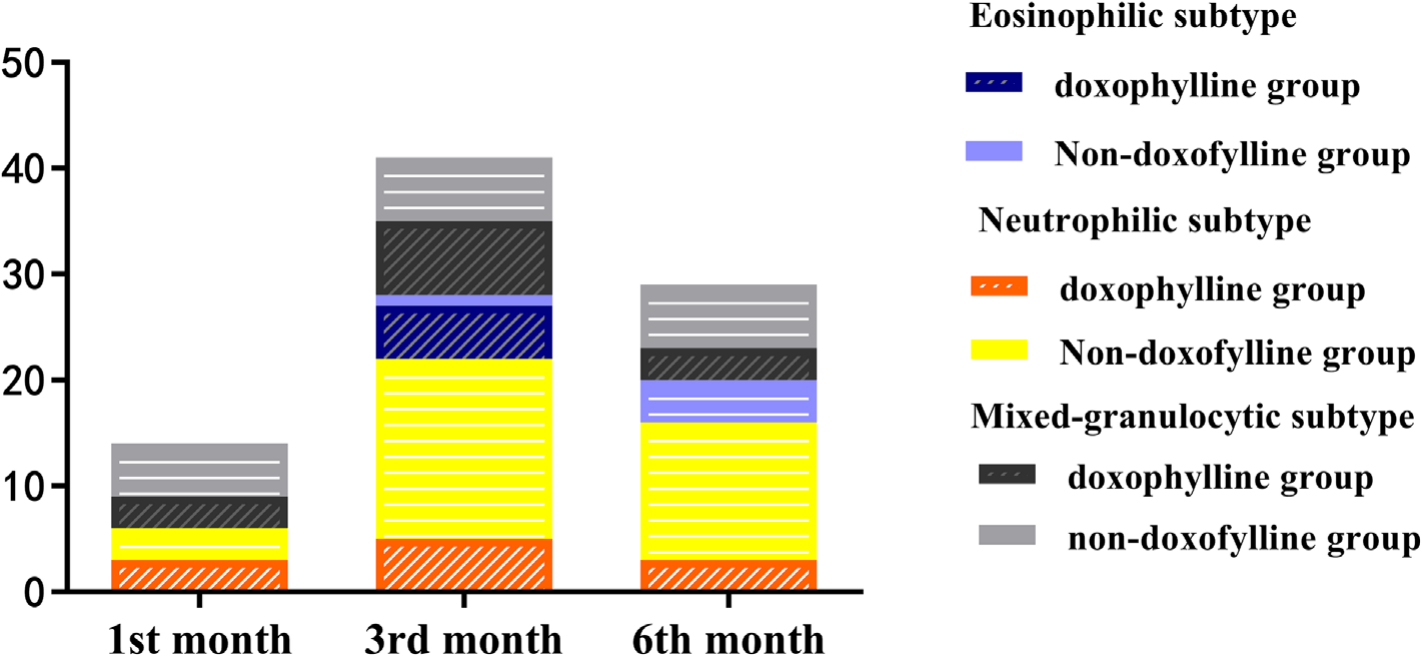
The incidence rates of exacerbations for each clinical subtype at the first, third, and sixth months compared with the baseline.

For the secondary outcomes of FEV_1_, CAT score, mMRC dyspnea score, and adverse events (COPD‐related and overall), there were no significant differences between the DG and the NDG for the three clinical subtypes. In the sixth month after discharge, results for inflammatory cells in the serum and post‐bronchodilator spirometry were collected and compared with the stable stage. For the eosinophilic subtype, the FEV_1_ and maximal mid‐expiratory flow (MEF) levels in the DG were significantly higher than those in the NDG (55.0% vs. 46.5%; 45.0% vs. 34.0%; *P* > 0.05), but the difference was not statistically significant. The mean neutrophil percentages were significantly different in the neutrophilic subtype (62.9% vs. 66.8%; *P* = 0.023) and the eosinophilic subtype (55.1% vs. 60.9%; *P* = 0.017), and the eosinophil percentages were significantly different in the neutrophilic subtype (1.0% vs. 1.8%; *P* = 0.009). The incidence rates of exacerbations for each clinical subtype at the first, third, and sixth months compared with the baseline are presented in Tables 3 and 4 and Figure 3.

**TABLE 3:** Outcomes for participants to doxophylline group and non‐doxophylline group, clinical phenotypes, per‐protocol population.

Primary outcomes	Neutrophilic		Eosinophilic		Mixed granulocytic	
	Doxophylline group	Non‐doxophylline group	Doxophylline group	Non‐doxophylline group	Doxophylline group	Non‐doxophylline group
Number of severe exacerbations (*n*, %)						
1 month after discharge	3 (3.9%)	3 (3.9%)	0 (0.0%)	0 (0.0%)	2 (3.8%)	3 (5.8%)
Incidence rate ratio (95% CI)	1.3 (0.3–6.0)		‐		1.1 (0.2–5.8)	
*P* value	0.529		‐		0.941	
3 months after discharge	5 (6.6%)	17 (22.4%)^a^	5 (18.5%)	1 (3.7%)	7 (13.5%)	6 (11.5%)
Incidence rate ratio (95% CI)	0.4 (0.2–0.9)		4.00 (0.54–29.81)		1.9 (0.7–4.8)	
*P* value	0.024		0.182		0.188	
6 months after discharge	3 (3.9%)	13 (17.1%)^a^	0 (0.0%)	4 (14.8%)^a^	3 (5.8%)	6 (11.5%)
Incidence rate ratio (95% CI)	0.3 (0.1–0.9)		1.5 (1.0–2.2)		0.8 (0.2–2.8)	
*P* value	0.045		0.028		0.728	

^a^ There was a clinically significant difference between DG and NDG.

**TABLE 4:** Outcomes for participants to doxophylline group and non‐doxophylline group, clinical phenotypes, per‐protocol population.

Secondary outcomes	Neutrophilic		Eosinophilic		Mixed granulocytic		*P* value
	Doxophylline group	Non‐doxophylline group	Doxophylline group	Non‐doxophylline group	Doxophylline group	Non‐doxophylline group	
Time of severe exacerbations (*n*, %)							
1 month	0.1 ± 0.3	0.1 ± 0.3	0.0 ± 0.0	0.0 ± 0.0	0.1 ± 0.3	0.1 ± 0.3	0.738
3 months	0.1 ± 0.4	0.4 ± 0.5	0.3 ± 0.5	0.1 ± 0.3	0.4 ± 0.5	0.2 ± 0.4	0.075
6 months	0.1 ± 0.3	0.4 ± 0.6	0.0 ± 0.0	0.4 ± 0.7	0.2 ± 0.5	0.2 ± 0.5	0.05
Total time	0.3 ± 0.5	0.9 ± 0.6^a^	0.3 ± 0.5	0.5 ± 0.7^a^	0.7 ± 0.6	0.5 ± 0.6	0.001
FEV_1_% predicted	41.0	46.0	55.0	46.5	46.0	46.0	0.304
MEF%	28.0	34.0	45.0	34.0	35.5	36.0	0.321
Leukocyte (10^9/L)‐6 months	7.6 ± 2.9	7.9 ± 2.8	6.1 ± 2.0	7.1 ± 2.2	6.4 ± 2.0	7.3 ± 2.0	0.148
Neutrophil percentage (%)	62.9	66.8^a^	55.1	60.9^a^	63.7	65.6	<0.001
Lymphocyte percentage (%)	18.2	23.1^a^	28.1	25.7^a^	20.1	23.7	0.027
Eosinophil percentage (%)	1.0	1.8^a^	4.2	4.6^a^	1.4	2.7^a^	<0.001

^a^ There was a clinically significant difference between DG and NDG.

### 3.3 Dose adjustment rate of inhaled drugs containing glucocorticoid

One hundred twenty‐five participants were treated with inhaled drugs containing glucocorticoid during the 6 months of follow‐up, comprising 54 participants in the DG and 71 in the NDG, and there was no significant difference in use of these drugs between the two groups (*P* > 0.05). During the follow‐up period, 42 patients in the DG reduced their dose of inhaled corticosteroids because the disease was well controlled or stable, and 24 patients increased their dose because of disease aggravation. In the NDG, 39 patients reduced their dose of inhaled corticosteroids, whereas 46 increased their dose. However, in the first and third months, there were no significant differences in the dose adjustment rate of inhaled drugs containing corticosteroids between the two groups (*P* > 0.05). In the sixth month, 25 patients in the DG reported reduced frequency of inhaled glucocorticoid, whereas six participants had increased use. In the NDG, 20 participants had reduced and 19 participants had increased the frequency of their use of inhaled glucocorticoid. The difference between the two groups was statistically significant (*P* = 0.033). The dose adjustment rates of inhaled drugs containing glucocorticoid for the DG and the NDG are presented in Table 5.

**TABLE 5:** The dose adjustment rate of inhaled drugs containing glucocorticoid between doxophylline group and non‐doxophylline group.

Dose adjustment rate (*n*, %)		Doxophylline group (*n* = 54)	Non‐doxophylline group (*n* = 71)	Total	*P* value
1st month	Reduced	0 (0.0%)	0 (0.0%)	0 (0.0%)	0.722
	Increased	4 (3.2%)	6 (4.8%)	10 (8.0%)	
3rd months	Reduced	16 (12.8%)	24 (15.2%)	35 (28.0%)	0.921
	Increased	14 (11.2%)	21 (16.8%)	35 (28.0%)	
6th months	Reduced	25 (20.0%)	20 (16.0%)	45 (36.0%)	0.037
	Increased	6 (4.8%)	19 (15.2%)	25 (20.0%)	

For the neutrophilic subtype, 25 participants in the DG and 35 in the NDG were treated with inhaled drugs containing glucocorticoids. There was no significant difference in the dose adjustment rate between the two groups in the first month (*P* > 0.05). In the third month, 10 participants had reduced and two had increased their dose in the DG, whereas in the NDG, seven participants had reduced and 17 had increased their dose. There was thus a significant difference between the two groups (*P* = 0.003). In the sixth month, in the DG, 13 participants had reduced and three had increased their dose, whereas in the NDG, six participants had reduced and nine had increased their dose, representing a significant difference between the two groups (*P* = 0.016).

For the eosinophilic subtype, 10 participants in the DG and 11 in the NDG were treated with inhaled drugs containing glucocorticoids. There was no significant difference in the dose adjustment rate between the two groups in the first‐ or third‐month follow‐ups (*P* > 0.05). In the sixth month, seven participants had reduced and 0 had increased their dose in the DG, whereas in the NDG, one participant had reduced and four had increased their dose. There was thus a significant difference between the two groups (*P* = 0.007).

For the mixed granulocytic subtype, 18 participants in the DG and 26 in the NDG were treated with inhaled drugs containing glucocorticoids. However, there were no significant differences in the dose adjustment rate among this subtype during the 6‐month follow‐up period (*P* > 0.05). The rates of dose adjustment of inhaled drugs containing glucocorticoid for each of the clinical phenotypes are presented in Table 6.

**TABLE 6:** The dose adjustment rate of inhaled drugs containing glucocorticoid of clinical phenotypes.

Dose adjustment rate (*n*,%)		Neutrophilic		Eosinophilic		Mixed granulocytic	
		Doxophylline group	Non‐doxophylline group	Doxophylline group	Non‐doxophylline group	Doxophylline group	Non‐doxophylline group
1st month	Reduced	0 (0.0%)	0 (0.0%)	1 (4.8%)	0 (0.0%)	0 (0.0%)	0 (0.0%)
	Increased	2 (3.3%)	3 (5.0%)	0 (0.0%)	0 (0.0%)	2 (4.5%)	3 (6.8%)
*P* Value		0.990		0.476		0.990	
3rd months	Reduced	10 (16.7%)	7 (11.7%)	1 (4.8%)	8 (38.1%)	5 (11.4%)	7 (15.9%)
	Increased	2 (3.3%)	17 (28.3%)	4 (40.0%)	1 (9.1%)	7 (15.9%)	4 (9.1%)
*P* Value		0.003		0.016		0.170	
6th months	Reduced	13 (21.7%)	6 (10.0%)	7 (33.3%)	1 (4.8%)	5 (11.4%)	10 (22.7%)
	Increased	3 (5.0%)	9 (15.0%)	0 (0.0%)	4 (19.0%)	3 (6.8%)	6 (13.6%)
*P* value		0.016		0.007		0.572	

## 4 DISCUSSION

The occurrence of COPD is determined by both environmental and genetic factors, and it is closely related to chronic inflammation, oxidative stress, imbalance of protease and antiprotease, apoptosis, and so on. The pathogenesis and pathological changes of the condition are complex and heterogeneous.^15^ Acute aggravated hospitalization and stable long‐term maintenance treatment are the main sources of medical burden, and efficacy, safety, individualization, and high drug prices are the urgent problems to be solved in the treatment of COPD.

In this prospective cohort study, patients with the clinical subtypes of neutrophilic and eosinophilic COPD who were treated with doxofylline in addition to an inhaled bronchodilator were found to be less likely to have severe exacerbations than patients treated with the inhaled bronchodilator only. Graham Devereux previously found that patients with bronchial asthma experienced the greatest benefit after using doxofylline for 6 weeks, whereas patients with COPD observed the greatest benefit from the eighth week.^16^

Among the neutrophilic subtype, the mean number of patients with exacerbations was lower in the DG than in the NDG in the third month after discharge (6.6% vs. 22.4%), showing a 15.8% reduction in the risk of severe exacerbations. In the sixth month after discharge, a 13.2% reduction was seen (3.9% vs. 17.1%). Among the eosinophilic subtype, the mean number of patients with exacerbations was lower in the DG than in the NDG in the sixth month after discharge (0.0% vs. 14.8%), showing a 14.8% reduction in the risk of severe exacerbations. According to the current trial, theophylline can reduce the number of severe COPD exacerbations requiring hospital admission, with the most benefit being evident in the subgroup of those patients frequently hospitalized with COPD.^17^ This differs from the results of a study published in 2019.^18^ It is unknown whether this may be because of the use of a low‐dose treatment.

COPD is a condition involving chronic inflammation of the airway that always occurs repeatedly and develops progressively, resulting in the remodeling of the airway structure and the destruction of the alveolar structure. It has been found that the main airway cells involved in the inflammation include activated neutrophils, macrophages, eosinophils, and lymphocytes.^19^ Of these, the levels of neutrophils and eosinophils are closely related to acute exacerbation and deterioration of COPD.^20^, ^21^ As one of the most important phenotypes of COPD, the inflammatory phenotype is advantageous for assessing acute exacerbation of COPD and evaluating prognosis. The inflammatory response is closely related to inflammatory markers, which can be expressed more accurately. In this study, the mean levels of neutrophils and eosinophils were significantly lower in the DG than in the NDG (62.9% vs. 66.8% and 1.0% vs. 1.8%, respectively) in the neutrophilic clinical subtype, and the neutrophil level was significantly lower in the DG than in the NDG (55.1% vs. 60.9%) in the eosinophilic subtype. These findings are similar to the results presented by Page and Culpitt.^22^, ^23^ In contrast, for the mixed granulocytic clinical subtype, there were no significant differences between inflammation cell percentages. Our results support that doxofylline can reduce acute aggravation and deterioration of neutrophilic and eosinophilic clinical subtypes and reduce airway inflammation. Other researchers using doxofylline in the treatment of COPD have found that it can increase the release of IL‐10 and exhibit an anti‐inflammatory and immunomodulatory effect, inhibit the release of inflammatory mediators by mast cells and the oxygen reactive of neutrophils,^24^ inhibit the translocation of proinflammatory transcription factor nuclear factor B (NF‐κB) into the nucleus and reduce the expression of inflammatory genes,^25^ and promote the apoptosis of neutrophils in vitro by reducing the anti‐apoptotic protein Bcl‐2.^26^ Doxofylline can decrease the recruitment of airway inflammatory cells and the release of inflammatory mediators; reduce leukocytes, neutrophils, and eosinophils in a variety of ways; and reduce airway inflammation and hyperresponsiveness in patients with COPD. In a study by Rajanandh and a pharmacological trial in Italy, the use of corticosteroids was shown to be lower in patients who took doxofylline as part of their respiratory disease treatments than those who did not.^27^, ^28^

In the current study, 125 of the participants were treated with inhaled drugs containing glucocorticoid during the 6‐month follow‐up period. In the sixth month, there was a significant difference between the dose adjustment rates of inhaled drugs containing glucocorticoid for the DG and the NDG (20.0% vs. 16.0% reduced, 4.8% vs. 15.2% increased; *P* = 0.033). In the third month after discharge, there was a significant difference in the adjustment rates among the neutrophilic subtype (16.7% vs. 11.7% reduced, 3.3% vs. 28.3% increased; *P* = 0.019). In the sixth month, the drug dose adjustment rates were 21.7% vs. 10.0% reduced and 5.0% vs. 15.0% increased (*P* = 0.016). For the eosinophilic subtype, there was also a significant difference in the sixth month (33.3% vs. 4.8% reduced, 0.0% vs. 19.0% increased; *P* = 0.019). However, there were no significant differences in dose adjustment rates for the mixed granulocytic clinical subtype during the 6‐month follow‐up period (*P* > 0.05). The rate of increasing drug dose in the DG was significantly lower than that in the NDG. This shows that the reported doses of inhaled drugs containing glucocorticoid for the neutrophil and eosinophil subtypes were significantly lower in the DG than that in the NDG, which supports that the use of doxofylline as an adjuvant therapy can reduce the demand for corticosteroids. Ford^29^ found that theophylline can reduce the dosage of glucocorticoids and improve corticosteroid resistance in patients with COPD. The combined use of these drugs has anti‐inflammatory effects, which can synergistically induce and enhance the responsiveness of steroid hormones to reduce the dosage of corticosteroids.^30^ This may be related to the fact that theophylline can activate histone deacetyl 2 in the macrophages of patients with COPD,^28^ restoring its activity to normal levels to increase glucocorticoid sensitivity and reverse corticosteroid resistance. At the same time, it works together with glucocorticoids to enhance the transcription of inflammatory cell genes and reduce the synthesis of pro‐inflammatory mediators.^31^

Pulmonary function is an important and intuitive index for measuring airflow limitation with good repeatability. It has great significance in the diagnosis, severity evaluation, disease progression, prognosis, and treatment response of COPD.^32^ In this study, when comparing the changes of pulmonary function between the stable stage and the sixth month after discharge, we found an interesting phenomenon: FEV_1_ and MEF levels in the DG for eosinophilic COPD were higher than for the other subtypes, but there were no significant differences between the three clinical subtypes. This suggests that doxofylline can delay the decline of pulmonary function and that the protective effect on pulmonary function in patients with eosinophilic COPD is better than that for the neutrophilic and mixed granulocytic subtypes, which is similar to the findings presented by Lal.^33^, ^34^ MEF is mainly determined by the non‐force‐dependent part of FVC, which can reflect the severity of airway obstruction and indicate the respiratory reserve strength, muscle strength, and dynamic level of a patient. Related studies have found that doxofylline has a direct relaxing effect on bronchial smooth muscle.^35^ It can strengthen the contractile force of the ventilator and eliminate ventilator fatigue,^36^ promote the movement of airway cilia, enhance the speed of mucociliary transport, and remove airway secretions.^37^

Drug safety is one of the core issues in the treatment of COPD. Theophylline is mainly metabolized through the cytochrome P450 microsomal enzyme system of CYP1A2 in the liver.^38^ Doxofylline lacks the ability to interfere with the cytochrome enzymes CYP1A2, CYP2E1, and CYP3A4, which prevents the significant interaction of other drugs metabolized in the liver through these pathways to produce a stable serum concentration.^39^ Recent pharmacological studies have shown that doxofylline does not directly inhibit any HDAC enzyme or any PDE enzyme subtype, nor does it antagonize any known adenosine receptors. This may explain why the safety of doxofylline has been improved.^40^ During the 1‐week drug adaptation period at the beginning of the current study, in the DG, acid regurgitation occurred in two patients (2.9%), who each had a history of chronic gastritis, and palpitation occurred in three patients (5.9%) with a history of arrhythmia. This suggests that doxofylline should be used cautiously in patients with chronic gastritis or peptic ulcers and arrhythmia. During the 6‐month follow‐up period, there were no serious adverse reactions among the DG, indicating that the incidence of adverse reactions is very low and the safety of clinical use is high.

### 4.1 Advantages and limitations

The design of this study had several advantages. First, unified standards and procedures were adopted and implemented by a respiratory professional attending physician with more than 5 years of working experience and skilled knowledge of how to operate the pulmonary function meter. Second, the data collection was jointly undertaken by two researchers to ensure that the observation forms were filled out in a detailed and objective manner. All the survey data and experimental data collected were input into the Epidata database. After data entry, consistency testing (comparison of differences after double entry of the questionnaire) and reliability testing (quality control of the input REC files) were carried out. Third, the researchers working on the study did not participate in the formulation of treatment plans. For follow‐up on the basis of chronic disease management, the participants had a high degree of fit, and the follow‐up intervals were designed to mitigate the risk of patients forgetting and thus control the rate of loss of follow‐up and improve the compliance of participants.

In terms of limitations, the research was a single‐center study, so the disease severity distribution and treatment of the study population may not represent the general disease population. In addition, the follow‐up period was 6 months, which is relatively short, and it is difficult to decide on causal effects about the changes of the level of the cell types. Finally, in consideration of medical accessibility and the disease treatment burden, the effects of doxofylline on mild and moderate acute exacerbation were not quantified.

## 5 CONCLUSIONS

According to the findings of a 6‐month follow‐up study, doxofylline, when used as an adjuvant drug, displays a favorable therapeutic effect on COPD patients with neutrophilic and eosinophilic clinical subtypes. It is capable of minimizing severe acute exacerbations, and in the case of neutrophilic subtypes, a consistent reduction in the number of exacerbations was observed after 3 months of use, whereas eosinophilic subtypes displayed a consistent reduction in exacerbations after 6 months, although further long‐term studies may be necessary to establish a concrete causal relationship. In addition, doxofylline can reduce reliance on glucocorticoids and promote long‐term reduction of inflammation (after at least 3 months of usage). Moreover, the incidence of adverse reactions associated with its use is low, making it a safe choice for clinical treatment.

## AUTHOR CONTRIBUTIONS

Mei–Feng Chen: the conception and design of the study; acquisition of data; drafting of the article. Wei He: defining the cases and revising them critically for important intellectual content. De‐Sheng Huang: analysis and interpretation of data. Hui Jia, Zhao‐Shuang Zhong, Nan Li, Shan‐Shan Li: acquisition of data. Shu‐yue Xia: revising them critically for important intellectual content; final approval of the version to be submitted.

## CONFLICT OF INTEREST STATEMENT

The authors declare that they have no competing interests.

## ETHICS STATEMENT

This study was conducted in line with the Helsinki Statement and approved by the Ethics Committee of Shenyang Medical College Hospital. All patients provided written informed consent before undertaking the study.

## Data Availability

The data that support the findings of this study are available from the corresponding author upon reasonable request.

## References

[1] Wang C , Xu J , Yang L , et al. Prevalence and risk factors of chronic obstructive pulmonary disease in China (the China Pulmonary Health[CPH]study): a national cross‐sectional study. The Lancet. 2018;391(10131):1706‐1717. doi:10.1016/S0140-6736(18)30841-9 29650248

[2] WHO . Disease Burden and Mortality Estimates CAUSE‐SPECIFIC MORTALITY, 2000–2016[R/OL].

[3] Cazzola M , Rogliani P , Puxeddu E , Ora J , Matera MG . An overview of the current management of chronic obstructive pulmonary disease: can we go beyond the GOLD recommendations. Expert Rev Respir Med. 2018;12(1):43‐54. doi:10.1080/17476348.2018.1398086 29082808

[4] Camiciottoli G , Bigazzi F , Paoletti M , Cestelli L , Lavorini F , Pistolesi M . Pulmonary function and sputum characteristics predict computed tomography phenotype and severity of COPD. Eur Respir J. 2013;42(3):626‐635. doi:10.1183/09031936.00133112 23258785

[5] Sidhaye VK , Nishida K , Martinez FJ . Precision medicine in COPD: where are we and where do we need to go? Eur Respir Rev. 2018;27(149):180022. Published 2018 Aug 1. doi:10.1183/16000617.0022-2018 30068688 PMC6156790

[6] Han MK , Agusti A , Calverley PM , et al. Chronic obstructive pulmonary disease phenotypes: the future of COPD. Am J Respir Crit Care Med. 2010;182(5):598‐604. doi:10.1164/rccm.200912-1843CC 20522794 PMC6850732

[7] Wedzicha JA , Singh D , Tsiligianni I , et al. Treatment response to indacaterol/glycopyrronium versus salmeterol/fluticasone in exacerbating COPD patients by gender: a post‐hoc analysis in the FLAME study. Respir Res. 2019;20(1):4. doi:10.1186/s12931-019-0972-7 30621717 PMC6325763

[8] 2022 Global Strategy for Prevention, Diagnosis and Management of COPD.

[9] Shukla D , Chakraborty S , Singh S , Mishra B . Doxofylline: a promising methylxanthine derivative for the treatment of asthma and chronic obstructive pulmonary disease. Expert Opin Pharmacother. 2009;10(14):2343‐2356. doi:10.1517/14656560903200667 19678793

[10] Cruse G , Duffy SM , Brightling CE , Bradding P . Functional KCa 3.1K+ channels are required for human lung mast cell migration. Thorax. 2006;61(10):880‐885. doi:10.1136/thx.2006.060319 16809411 PMC2104766

[11] Cazzola M , Calzetta L , Rogliani P , Page C , Matera MG . Impact of doxofylline in COPD: a pairwise meta‐analysis. Pulm Pharmacol Ther. 2018;51:1‐9. doi:10.1016/j.pupt.2018.04.010 29705620

[12] Riffo‐Vasquez Y , Venkatasamy R , Page CP . Steroid sparing effects of doxofylline. Pulm Pharmacol Ther. 2018;48:1‐4. doi:10.1016/j.pupt.2017.10.008 29031617

[13] CAT Governance Board . COPD Assessment Test 2016. Accessed July 2, 2018. http://www.catestonline.org/

[14] Fletcher CM . Standardised questionnaire on respiratory symptoms. BMJ. 1960;2(5213):1665. doi:10.1136/bmj.2.5213.1665 13688719

[15] Barnes PJ . Theophylline: new perspectives on an old drug. Am J Respir Crit Care Med. 2003;167(6):813‐818. doi:10.1164/rccm.200210-1142PP 12623857

[16] Ge JB , Xu YJ , Wang C . Internal Medicine. People's Medical Publishing House; 2018.

[17] Devereux G , Cotton S , Fielding S , et al. Effect of theophylline as adjunct to inhaled corticosteroids on exacerbations in patients with COPD: a randomized clinical trial. Jama. 2018;320(15):1548‐1559. doi:10.1001/jama.2018.14432 30326124 PMC6233797

[18] Devereux G , Cotton S , Fielding S , et al. Low‐dose oral theophylline combined with inhaled corticosteroids for people with chronic obstructive pulmonary disease and high risk of exacerbations: a RCT. Health Technol Assess. 2019;23(37):1‐146. doi:10.3310/hta23370 PMC6689818 31343402

[19] Avci E , Avci AG . Important biomarkers that play a role in the chronic obstructive pulmonary disease process. J Med Biochem. 2018;37(1):46‐53. doi:10.1515/jomb-2017-0035 30581341 PMC6294106

[20] Vedel‐Krogh S , Nielsen SF , Lange P , Vestbo J , Nordestgaard BG . Blood eosinophils and exacerbations in chronic obstructive pulmonary disease. The Copenhagen general population study. Am J Respir Crit Care Med. 2016;193(9):965‐974. doi:10.1164/rccm.201509-1869OC 26641631

[21] Yun JH , Lamb A , Chase R , et al. Blood eosinophil count thresholds and exacerbations in patients with chronic obstructive pulmonary disease. J Allergy Clin Immunol. 2018;141(6):2037‐2047. doi:10.1016/j.jaci.2018.04.010 29709670 PMC5994197

[22] Page C , Cazzola M . Bifunctional drugs for the treatment of asthma and chronic obstructive pulmonary disease. Eur Respir J. 2014;44(2):475‐482. doi:10.1183/09031936.00003814 24696121

[23] Culpitt SV , de Matos C , Russell RE , Donnelly LE , Rogers DF , Barnes PJ . Effect of theophylline on induced sputum inflammatory indices and neutrophil chemotaxis in chronic obstructive pulmonary disease. Am J Respir Crit Care Med. 2002;165(10):1371‐1376. doi:10.1164/rccm.2105106 12016098

[24] Şahin F , Koşar AF , Aslan AF , Yiğitbaş B , Uslu B . Serum biomarkers in patients with stable and acute exacerbation of chronic obstructive pulmonary disease: a comparative study. J Med Biochem. 2019;38(4):503‐511. Published 2019 Jan 22. doi:10.2478/jomb-2018-0050 31496916 PMC6708295

[25] Ichiyama T , Hasegawa S , Matsubara T , Hayashi T , Furukawa S . Theophylline inhibits NF‐κ B activation and IκB alpha degradation in human pulmonary epithelial cells. Naunyn Schmiedebergs Arch Pharmacol. 2001;364(6):558‐561. doi:10.1007/s00210-001-0494-x 11770011

[26] Chung IY , Nam‐Kung EK , Lee NM , et al. The downregulation of bcl‐2 expression is necessary for theophylline‐induced apoptosis of eosinophil. Cell Immunol. 2000;203(2):95‐102. doi:10.1006/cimm.2000.1678 11006007

[27] Rajanandh MG , Nageswari AD , Ilango K . Pulmonary function assessment in mild to moderate persistent asthma patients receiving montelukast, doxofylline, and tiotropium with budesonide: a randomized controlled study. Clin Ther. 2014;36(4):526‐533. doi:10.1016/j.clinthera.2014.02.006 24650447

[28] Mennini FS , Sciattella P , Marcellusi A , Marcobelli A , Russo A , Caputi AP . Treatment plan comparison in acute and chronic respiratory tract diseases: an observational study of doxophylline vs. theophylline. Expert Rev Pharmacoecon Outcomes Res. 2017;17(5):1‐8. doi:10.1080/14737167.2017.1301815 28277853

[29] Ford PA , Durham AL , Russell REK , Gordon F , Adcock IM , Barnes PJ . Treatment effects of low‐dose theophylline combined with an inhaled corticosteroid in COPD. Chest. 2010 Jun;137(6):1338‐1344. doi:10.1378/chest.09-2363 20299628

[30] Rajanandh MG , Nageswari AD , Ilango K . Assessment of various second‐line medications in addition to inhaled corticosteroid in asthma patients: a randomized controlled trial. Clin Exp Pharmacol Physiol. 2014;41(7):509‐513. doi:10.1111/1440-1681.12239 24738981

[31] Cosio BG , Tsaprouni L , Ito K , Jazrawi E , Adcock IM , Barnes PJ . Theophylline restores histone deacetylase activity and steroid responses in COPD macrophages. J Exp Med. 2004;200(5):689‐695. doi:10.1084/jem.20040416 15337792 PMC2212744

[32] Chinese Medical Association . Guideline for primary care of chronic obstructive pulmonary disease(2018). CMA. 2018;17(11):856‐870. doi:10.3760/cma.j.issn.1671-7368

[33] Lal D , Manocha S , Ray A , Vijayan VK , Kumar R . Comparative study of the efficacy and safety of theophylline and doxofylline in patients with bronchial asthma and chronic obstructive pulmonary disease. J Basic Clin Physiol Pharmacol. 2015;26(5):443‐451. doi:10.1515/jbcpp-2015-0006 25894641

[34] Ram FS , Jones PW , Castro AA , et al. Oral theophylline for chronic obstructive pulmonary disease. Cochrane Database Syst Rev. 2002;2002(4):CD003902. doi:10.1002/14651858.CD003902 PMC7047557 12519617

[35] Wan XH , Lu XF . Diagnostics. People's Medical Publishing House; 2018.

[36] Cazzola M , Calzetta L , Barnes PJ , et al. Efficacy and safety profile of xanthines in COPD: a network meta‐analysis. Eur Respir Rev. 2018;27(148):180010. doi:10.1183/16000617.0010-2018 29720510 PMC9488859

[37] Spina D , Page CP . Xanthines and phosphodiesterase inhibitors. Handb Exp Pharmacol. 2017;237:63‐91. doi:10.1007/164-2016-71 27844172

[38] Sarkar MA , Hunt C , Guzelian PS , Karnes HT . Characterization of human liver cytochromes P‐450 involved in theophylline metabolism. Drug Metab Dispos. 1992;20(1):31‐37. 1346993

[39] Sankar J , Lodha R , Kabra SK . Doxophylline: the next generation methylxanthine. The Indian Journal of Pediatrics. 2008;75(3):251‐254. doi:10.1007/s12098-008-0054-1 18376093

[40] Van Mastbergen J , Jolas T , Allegra L , et al. The mechanism of action of doxofylline is unrelated to HDAC inhibition, PDE inhibition or adenosine receptor antagonism. Pulmonary Pharmacology and Therapeutics. 2012;25(1):55‐61. doi:10.1016/j.pupt.2011.10.007 22138191

